# The role of cholesterol metabolism in lung cancer

**DOI:** 10.32604/or.2024.047933

**Published:** 2024-09-18

**Authors:** WEIGANG XIU, XINGYU LIU, KAIXIN HU, QIN ZHANG, HUASHAN SHI

**Affiliations:** 1 Department of Thoracic Oncology and State Key Laboratory of Biotherapy, Cancer Center, West China Hospital, Sichuan University, Chengdu, 610041, China; 2 West China School of Medicine, Sichuan University, Chengdu, 610041, China; 3 Department of Postgraduate Students, West China School of Medicine, Chengdu, 610041, China; 4 Department of Biotherapy, Cancer Center, West China Hospital, Sichuan University, Chengdu, 610041, China

**Keywords:** Cholesterol metabolism, Lung cancer, Immune cells, Targeted strategies

## Abstract

Elevated serum cholesterol metabolism is associated with a reduced risk of lung cancer. Disrupted cholesterol metabolism is evident in both lung cancer patients and tumor cells. Inhibiting tumor cell cholesterol uptake or biosynthesis pathways, through the modulation of receptors and enzymes such as liver X receptor and sterol-regulatory element binding protein 2, effectively restrains lung tumor growth. Similarly, promoting cholesterol excretion yields comparable effects. Cholesterol metabolites, including oxysterols and isoprenoids, play a crucial role in regulating cholesterol metabolism within tumor cells, consequently impacting cancer progression. In lung cancer patients, both the cholesterol levels in the tumor microenvironment and within tumor cells significantly influence cell growth, proliferation, and metastasis. The effects of cholesterol metabolism are further mediated by the reprogramming of immune cells such as T cells, B cells, macrophages, myeloid-derived suppressor cells, among others. Ongoing research is investigating drugs targeting cholesterol metabolism for clinical treatments. Statins, targeting the cholesterol biosynthesis pathway, are widely employed in lung cancer treatment, either as standalone agents or in combination with other drugs. Additionally, drugs focusing on cholesterol transportation have shown promise as effective therapies for lung cancer. In this review, we summarized current research regarding the rule of cholesterol metabolism and therapeutic advances in lung cancer.

## Introduction

In recent years, a growing number of studies [1] have highlighted the significant impact of body metabolism on the initiation and progression of tumors. Tumor cells and immune cells exhibit rapid reprogramming in the tumor microenvironment (TME), influenced by the levels of metabolic substances such as carbohydrates, proteins, and lipids [2]. Furthermore, there has been increasing attention to the role of cholesterol metabolism in cancer. A previous study has demonstrated a correlation between serum cholesterol levels and progression, treatment effect and prognosis of cancer. Another study conducted in Australia suggested that higher total serum cholesterol was associated with a reduced risk of total cancer in men (hazard ratio [HR] = 0.94; 95% confidence interval, 95% [CI]: 0.88-1.00) [3]. This association may be attributed to the impact of preclinical cancer effect on cholesterol metabolism. Cancers in preclinical stages have the potential to impede the metabolism of serum cholesterol, leading to a decrease in serum cholesterol levels. For instance, chronic liver disease, a precursor to liver cancer, can impede cholesterol evacuation from tumor cells due to hepatic inadequacy, resulting in a reduction of serum cholesterol levels.

Lung cancer is one of the types with the highest occurrence and death rate, contributing to a 20% death rate associated with cancer [4]. In recent years, there has been significant attention on the relationship between cholesterol metabolism and lung cancer. Findings from a Korean cohort study indicated a negative correlation between serum cholesterol level and incidence rate of lung cancer in men (HR, 0.89, 95% CI, 0.82-0.96; *p* < 0.001), though no such correlation was observed in women, which may due to different lifestyle factors including smoking and eating habits [5]. Data from this large prospective study revealed that total cholesterol levels exhibit varying associations with cancer, depending on the specific cancer site. In patients with lung cancer, total cholesterol, low-density lipoprotein-cholesterol (LDL-C) and high-density lipoprotein-cholesterol (HDL-C) were significantly lower compared to the non-cancer group [6]. However, the underlying mechanisms were not elucidated in these two studies. Notably, in patients treated with immune checkpoint inhibitor therapy, an increase in total cholesterol was linked to longer overall survival (OS) (*p* = 0.036) [7]. Another study indicated that individuals with high serum cholesterol levels (>200 mg/dL) experienced longer OS (19.4 months *vs*. 5.5 months, *p* = 0.001) [8]. Cholesterol plays an important role in immunomodulation, functioning as both an energy resource and a component of cell membranes. It is indispensable for the activation of immune cells, including T cells, natural killer, macrophages, and others. Reduced cholesterol levels inhibit the proliferation and activation of T cells. The aim of this review is to uncover the role of cholesterol metabolism in lung cancer, encompassing the effects of cholesterol metabolic and transport pathways on lung cancer, the roles of cholesterol metabolites in lung cancer, the effects of cholesterol metabolism on immune cells associated with lung cancer, and potential strategies targeting cholesterol metabolism for lung cancer treatment.

### Overview of cholesterol metabolism in tumor cells

Lipids play a crucial role in cellular functions. Among them, cholesterol, a 27-carbon-lipid, is essential for cellular metabolism and tumor cells. Cholesterol serves as a vital component of the cell membrane, influencing material transport, regulating membrane fluidity and stability, and participating in signal transduction. The intricate process of cholesterol metabolism involves exogenous uptake, biosynthesis, excretion, and storage [9]. Cholesterol is derived from both exogenous uptake and biosynthesis. To minimize energy consumption in cholesterol biosynthesis, cells express various receptors to facilitate the transport of exogenous cholesterol. In the human body, cholesterol is primarily transported by low-density lipoprotein (LDL) and high-density lipoprotein (HDL). Following absorption in the gastrointestinal tract, cholesterol from food is transported as chylomicrons to the liver and then distributed throughout the body via LDL. Inside cells, LDL is transported by LDL receptors (LDLR) and undergoes hydrolysis into fatty acids and cholesterol by lysosomal enzymes, such as lysosomal acid lipase (LAL) [10]. Evidence indicates that elevated cholesterol levels in colon cancer cells may promote cancer progression [11]. In TME, HDL can reduce cholesterol levels in tumor cells, thereby reversing tumor immune escape and inhibiting angiogenesis [12]. The elimination of LAL suppresses immune rejection and permits the growth of human lung cancer cells in LAL-deficient mice, highlighting the essential role of LAL homeostasis in maintaining antitumor immunity [13]. Endogenous cholesterol synthesis initiates with acetyl CoA, progressing to the formation of mevalonic acid through the action of 3-hydroxy-3-methylglutarate monoacyl CoA (HMG-CoA), and eventually results in cholesterol through more than 20 enzymes. The pivotal enzyme in this process is 3-Hydroxy-3-methylglutary coenzyme A reductase (HMGCR), serving as the first-rate limiting enzyme in cholesterol biosynthesis. Statin drugs exert their effects on the growth of tumor cells by dysregulating the activity of this enzyme [14]. The key regulator of cholesterol metabolism, SREBP2, upregulates the expression of HMGCR and other enzymes in response to low cellular cholesterol levels, thereby enhancing endogenous cholesterol uptake. Cholesterol metabolism also involves excretion and storage. Under the influence of Acyl coenzyme A-cholesterol acyltransferase (ACAT1), excess cholesterol can be esterified into cholesterol esters and then stored within cells as lipid droplets [15].

Conversely, intracellular cholesterol accumulation, particularly hydroxysteroid, can activate LXR [16]. This activation facilitates the removal of excess cholesterol from cells via ATP-binding cassette transporter A1 (ABCA1), and G1 (ABCG1). Subsequently, cholesterol is transported to the liver by HDL, where it is either converted into bile acid or directly discharged from the intestine through bile [17]. The removal of LXR can lead to cholesterol accumulation in tumor cells, contributing to a subtype of non-small cell lung carcinoma (NSCLC) [18].

In conclusion, there are four metabolic pathways of cholesterol in both normal cells and tumor cells. However, mechanisms have been discovered to enhance cholesterol uptake and biosynthesis in tumor cells, while cholesterol removal is weakened leading to an accumulation of cholesterol in tumor cells, including lung cancer, compared to normal tissue. The detailed mechanisms are illustrated below.

### The impact of cholesterol metabolism and transport pathways on lung cancer

**Figure 1:**
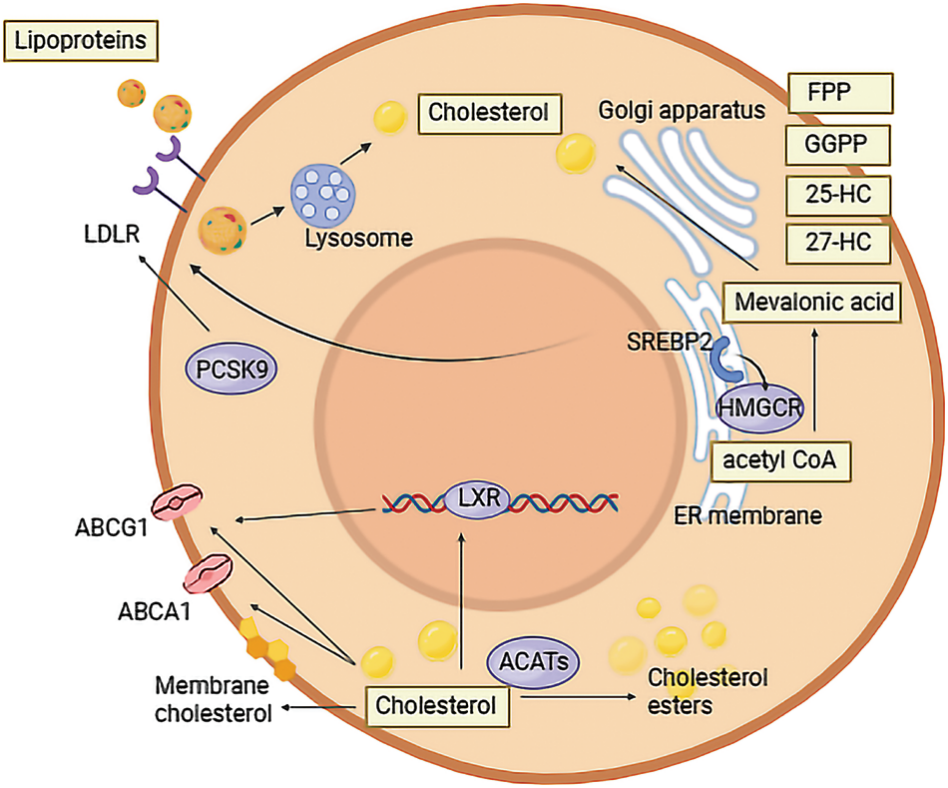
Cholesterol metabolism in tumor cells. In the form of LDL, cholesterol can be transported into tumor cells by LDLR, and then hydrolyzed into cholesterol. PCSK9 in tumor cells is a regulator of LDLR. Biosynthesis of cholesterol in tumor cells begins with acetyl CoA. It can be transformed into cholesterol by various enzymes, of which HMGCR is the most important. 25-HC, 27-HC, FPP and GGPP are metabolites of cholesterol biosynthesis. SREP2 can regulate HMGCR and LDLR expression in tumor cells. Apart from being esterified by ACATs and stored in cells, cholesterol can also be excreted by ABCG1 and ABCA1, under the regulation of LXR when cholesterol accumulates in tumor cells.

The cholesterol levels in tumor cells are generally recognized to be higher than in other tissues [19], indicating that factors involved in cholesterol metabolism and transport pathways can impact the progression of cancer. Favorably, factors that promote cholesterol uptake, biosynthesis and accumulation in tumor cells tend to support cancer progression. Conversely, components involved in mediating cholesterol efflux can inhibit cancer development. LXRs serve as key transcriptional regulators in cholesterol homeostasis [19]. Indeed, cholesterol accumulation in tumor cells can activate LXRs, thereby promoting cholesterol efflux via ABCA1 and ABCG1 [16]. Studies have demonstrated that LXR stimulation disrupts the AKT survival pathway, inducing apoptosis of tumor cells [20]. Interestingly, this apoptotic effect can be reversed by the addition of exogenous cholesterol. Conversely, inhibiting LXRs in tumor cells has been shown to decrease the expression of glycolytic and lipogenic genes, leading to apoptosis of tumor cells [21]. Inactivation of LXR genes results in cholesterol accumulation in lung cells, contributing to lung squamous cell carcinoma occurrence after 14 months [22]. This underscores the pivotal role of LXRs in preventing epithelial hyperplasia. Moreover, LXRαβ-/-mice develop a subtype of NSCLC at 18 months, characterized by a high proliferation rate of tumor cells [18]. A clinical study showed that higher LXRα levels correlate with significantly increased 5-year tumor-related survival rates in NSCLC patients (54% *vs*. 32%) [23]. ABCG1 promotes cholesterol efflux from tumor cells, thereby regulating intracellular cholesterol homeostasis [24], which is also a vital mediator of LXRs effects (Fig. 1). The upregulation of ABCG1 in lung cancer is associated with increased cancer cell proliferation, migration, and invasion, while inhibition by betulinic acid leads to G1 phase arrest, decreased proliferation, and migration potential of lung cancer cells [25,26].

Additionally, LDL plays a crucial role in facilitating cholesterol uptake by tumor cells, serving as a transporter of cholesterol from the liver to various tissues, including tumors. When cholesterol levels in tumor cells are low, SPEBP2 not only stimulates cholesterol biosynthesis but also promotes LDLR expression, thereby increasing cholesterol uptake. Research suggests that lower serum LDL cholesterol levels (<70 mg/dL) are associated with a significantly higher risk of lung cancer [27] and serum LDL levels are markedly lower in lung cancer patients compared to the control group. The pro-protein convertase subtilisin/kexin 9 (PCSK9) is a key regulator of cholesterol transport via LDL [28]. As a member of the pro-protein convertase family, PCSK9 can bind to LDLR, accelerating its degradation by lysosomes and consequently decreasing cholesterol uptake [29]. Research indicates that PCSK9 inhibitors can control the progression of non-cardiovascular diseases, including cancer [19]. This therapeutic effect may arise from the removal of the inhibitory impact on cholesterol uptake by tumor cells. In NSCLC, lower serum PCSK9 levels (<95 ng/mL) are associated with higher OS [30]. Moreover, NSCLC patients with a lower baseline serum PCSK9 exhibit longer progression-free survival and a better response to ICIs [31]. In lung adenocarcinoma (LAC) cells, transfection with PCSK9 small interfering (si) RNA induces mitochondrial dysfunction and apoptosis in tumor cells [32].

### Roles of cholesterol metabolites in lung cancer

Cholesterol derivatives, such as 25-hydroxy cholesterol (25-HC), 22-hydroxy cholesterol, 24-hydroxy cholesterol, and 27-hydroxy cholesterol (27-HC) have been identified as influential factors in cancer development [33]. Specifically, 25-HC and 27-HC are oxysterols generated as byproducts of cholesterol metabolism in various organs, including the lung [34]. Their significance in the progression of lung cancer has been established. Oxysterols contribute to the pro-cancerous effects of lung cancer through mechanisms such as inflammation or interactions with oxysterol-binding proteins [35]. Among oxysterols, 27-HC is the most prevalent type [36] and has been associated with the progression and metastasis of various tumors, including lung cancer [37]. In LAC, 27-HC is linked to elevated cholesterol levels and metastasis [38]. Mechanistically, 27-HC maintains cholesterol balance by promoting cholesterol execration via LXR and suppressing cholesterol production through the Insulin-induced Genes (INSIGs) [39]. Additionally, 27-HC can enhance the growth of end-stage cancers, potentially due to an imbalance in pro-survival and pro-inflammatory signals. Its effects involved the regulation of NFκB/PPIB axis and the secretion of FGF2 and IL-6. Research has shown that 27-HC can enhance osteoclast differentiation in conditioned media from LAC cells co-cultured with macrophages. This effect is induced by inhibiting miR-139 expression and activating the STAT3/c-Fos/NFATc1 pathway, ultimately leading to the inhibition of bone metastasis in LAC [40]. Additionally, 27-HC can inhibit cholesterol synthesis by suppressing HMGCR [41] and SREBP2 [42], consequently reducing cholesterol levels in tumor cells. 27-HC has been reported to have the ability to inhibit lung cancer progression, however, contrasting findings suggest that 25-HC can enhance it. 25-HC can decrease the sensitivity of tumor cells to 5-fluorouracil (5-FU), resulting in an increase in cell proliferation and decreased cell apoptosis when 5-FU is introduced [43]. Additionally, 25-HC has been shown to promote distant lung metastasis of tumor cells *in vivo*. As a ligand of LXR, 25-HC may impact the progression of lung cancer [44]. 25-HC was proven to promote the progression of LAC cells [45]. In the monoculture system, 25-HC promoted LAC cell migration and invasion in an LXR-dependent manner without affecting cell proliferation. However, in the co-culture system, 25-HC could stimulate interleukin-1β (IL-1β) secretion and increase the expression of LXR and Snail, enhancing the effect of 25-HC in an LXR-independent manner.

Isoprenoids, such as GGPP and farnesyl diphosphate (FPP), are also metabolites of cholesterol metabolism [46]. Cellular dysregulation in isoprenoid metabolism can be a contributing factor to cancer [47]. Geranylgeranyl diphosphate synthase (GGPPS) is the enzyme responsible for synthesizing GGPP from FPP, impacting the relative levels of FPP and GGPP in tumor cells [48]. Research has demonstrated a significantly higher expression of GGPPS in LAC tissues compared to adjacent normal tissues [49]. Moreover, the knockdown of GGPPS has been shown to inhibit the migration and invasion of LAC through the regulation of EMT due to the depletion of GGPP, while it does not affect cell proliferation and apoptosis [50].

### The effects of cholesterol metabolism on immune cells associated with lung cancer

During the progression of lung cancer, tumor cells continuously interact with surrounding stromal cells, including various immune cells, to form TME, exhibiting unique metabolic characteristics [51]. Tumor-infiltrating immune cells (TIICs) are key participants in TME, regulating the growth of tumor cells and displaying either anti-tumor or tumor-promoting functions [52]. Key components of TIICs include T cells, B cells, tumor-associated macrophages (TAMs), dendritic cells (DC), myeloid-derived suppressor cells (MDSCs), neutrophils and natural killer (NK) cells [53]. Cholesterol metabolism in the TME not only affects the functions of tumor cells, but can also reprogram the activities of TIICs, influencing immune functions.

MDSCs are heterogeneous, immature cell populations derived from the bone marrow that exert a negative effect on immune responses in autoimmune diseases, cancer, and chronic infections. They also play a crucial regulatory role in the invasion and metastasis of tumor cells, as well as in the generation of tumor blood vessels [54]. In the context of lung cancer, MDSCs, through interactions with inherent defense mechanisms, can cause indirect inflammatory damage to promote the progression of lung cancer. They can also restrain the anti-tumor ability of T cells. Research has shown that high serum cholesterol levels induce the generation and accumulation of MDSCs, promoting the rapid growth and spontaneous metastasis of primary tumors, including lung cancer, by inhibiting the activation of CD8+ T cells [55]. TAMs are an important class of natural immune cells in the TME. In lung cancer, TAMs can be divided into classic activation (M1) and alternative activation (M2) macrophages, exhibiting anti-tumor or tumor-promoting characteristics. TAMs can promote lung cancer progression through mechanisms such as promoting tumor angiogenesis, tumor cell metastasis, and immune escape [56]. In a study of LAC, there was a significant accumulation of cholesterol within tumor tissue. Interestingly, the cholesterol levels were found to be reduced in TAMs isolated from LAC tissues compared to adjacent normal tissues [57]. This indicates an opposite dysregulation of cholesterol homeostasis between tumor cells and TAMs. The research further suggests that cholesterol depletion in TAMs can have a distinct impact on macrophage gene expression, promoting angiogenesis, growth, and metastasis [57].

Cholesterol metabolism can affect other immune cells in lung cancer. Research indicates that a high serum cholesterol level can impair the anti-tumor ability of CD8+ T cells, and cholesterol accumulation in TME can induce cholesterol depletion in CD8+ T cells, compromising their anti-tumor functions [55]. In contrast, suppressing cholesterol esterification in T cells by inhibition of ACAT1 can increase the cholesterol level in the plasma membrane of CD8+ T cells. Consequently, this enhancement can promote the proliferation and signaling of CD8+ T cells, thereby controlling the progression of lung cancer [58]. Regulatory B cells restrict immune and inflammatory response in lung cancer. Research has shown that the synthesis of cholesterol metabolic intermediate GGPP is crucial for IL-10 production, which mediates the function of regulatory B cells [59]. NK cells, being cytotoxic lymphocytes of the innate immune system, possess the ability to eliminate tumor cells. Elevated serum cholesterol levels have been linked to an increase in the numbers of NK cells and the expression of NK cell-activating receptors, resulting in fewer tumor metastases in the lung [15]. The interplay between cholesterol levels and the immune response involving regulatory B cells and NK cells highlights the complexity of the immune landscape in the context of lung cancer.

### Targeting cholesterol metabolism for lung cancer strategies

It is well-established that alterations in cholesterol metabolism within tumor cells are considered one of the emerging hallmarks of cancer [60]. In recent years, there has been growing research into targeting cholesterol synthesis and transport processes to inhibit the occurrence and progression of various cancers, including lung cancer. These strategies primarily focus on the cholesterol biosynthesis pathway, particularly through HMGCR, such as statin drugs, and the transportation or delivery of cholesterol-modifying drugs.

Statins have been widely recognized as a safe and effective clinical anti-tumor approach. These drugs can inhibit HMGCR, a key enzyme involved in cholesterol biosynthesis. Consequently, they can lower LDL-C levels and elevate HDL-C levels. Originally identified as a fungal metabolite [61], statins gained prominence due to their early use as cholesterol-lowering drugs with minimal adverse effects. This led to their swift adoption in clinical oncology, including strategies for managing lung cancer [62]. In the realm of clinical therapy, research has shown that individuals receiving statins for more than 6 months before a lung cancer diagnosis had a 55% reduced risk of developing lung cancer [63]. In men with hypercholesterolemia, a high intake of statins is associated with a lower risk of lung cancer [64]. Furthermore, There is evidence that statin exposure is associated with significantly improved OS among patients with lung cancer [65]. Apart from the impact of cholesterol metabolism in tumor cells, statins were also found effective in regulating lung cancer TME in order to inhibit cancer progress and enhance treatment effects. Statins could reverse immuo-cold (tumors with limited tumor-infiltrating) TME to immuno-hot via cholesterol synthesis pathways in NSCLC, which significantly enhanced ICB efficacy [65].

The potential of combining statins with other anti-cancer therapies has also been explored [66], and statins have been found to be effective in overcoming resistance to epidermal growth factor receptor-tyrosine kinase inhibitors (EGFR-TKIs). The combination of statins with EGFR-TKIs therapy has been associated with prolonged OS in patients with lung cancer, indicating a synergistic anti-cancer effect [67]. Moreover, the combination of atorvastatin with EGFR-TKIs in the treatment of NSCLC demonstrated an enhanced anti-tumor effect *in vitro*, along with a mitigated growth of NSCLC *in vivo* [68]. Another study has shown that atorvastatin exhibits anti-tumor properties in NSCLC and can overcome resistance to EGFR-TKIs [69]. In NSCLC cells harboring an EGFR-resistant mutation, simvastatin could induce apoptotic cell death [70] (Table 1). Various drugs targeting the cholesterol transport process have shown promising results. For instance, when incorporated into cationic liposomes composed of DOTAP/cholesterol, all-trans retinoic acid (ATRA) exhibited significantly higher cytotoxic effects and apoptosis-induced activity [71]. Cepharanthine has been identified as an inhibitor of endolysosomal trafficking of cholesterol and LDL, enhancing the anti-tumor activity of the standard chemotherapy cisplatin in lung cancer [72]. Cholesterol can also serve as a medium for delivering drugs to lung cancer cells to promote anti-tumor effects. When a cholesterol-modified derivative of gemcitabine (Gem) is synthesized (Gem-Chol), it delays drug release and provides long-term stability, thereby improving the anti-tumor effect of Gem in the treatment of NSCLC [73]. In another study, a cholesterol-modified low molecular weight chitosan was employed as a delivery system for therapeutic small interfering RNA (siRNA) and a hydrophobic drug. This approach efficiently facilitated the uptake of promising siRNA by lung cancer as part of combination cancer therapy [74] (Table 2).

**Table 1:** Strategies targeting cholesterol biosynthesis pathway

Drugs	Subjects	Effects	Reference
Statins	US veterans	More than 6 months’ statins treatment could reduce the risk of lung cancer by 55%.	[63]
	Men with hypercholesterolemia	High statin intake could lower the risk of cancer.	[64]
	Patients with lung cancer	Statin exposure improved OS.	[65]
Statins combination with EGFR-TKIs	Patients with lung cancer	Statins and EGFR-TKIs played a synergistic anticancer role, which prolonged OS.	[67]
Atorvastatin in combination with EGFR-TKIs	NSCLC HCC827 cell line	Anti-tumor effect of EGFR-TKIs was enhanced *in vitro*. Growth of NSCLC was mitigated *in vivo*.	[68]
Atorvastatin in combination with EGFR-TKIs	NSCLC cells with EGFR-TKI resistance	Atorvastatin overcomes NSCLC with EGFR-TKIs resistance.	[69]
Simvastatin in combination with EGFR-TKIs	Gefitinib-sensitive (HCC827, E716-A750del) and-resistant (H1975, T790M + L858R) NSCLC cells	Simvastatin induced apoptotic cell death.	[70]

Note: US, United States; OS, overall survival; EGFR-TKIs, epidermal growth factor receptor-tyrosine kinase inhibitor; NSCLC, non-small cell lung carcinoma.

**Table 2:** Strategies targeting cholesterol transport process

Drugs	Subjects	Effects	Reference
ATRA incorporated in DOTAP/cholesterol	A549 human lung cancer cells	Higher cytotoxic effects and apoptosis-induced activity	[71]
Cepharanthine in combination with standard cisplatin chemotherapy	Mice with lung cancer	Enhanced the anti-tumor activity	[72]
Gem-Chol	H22 and S180 tumor xenograft Wistar male rats	Delayed drug release and long-term stability of Gem	[73]
Cholesterol-modified chitosan as a delivery system for therapeutic siRNA and a hydrophobic drug	A549 human lung cancer cells	Promised uptake and effect of siRNA as a combination therapy	[74]

Note: ATRA, all-trans retinoic acid; DOTAP, Gem-Chol gemcitabine-cholesterol; Gem, gemcitabine; siRNA, small interfering RNA.

## Conclusions and Prospects

In recent years, there has been a growing recognition of the significance of cholesterol metabolism in the context of lung cancer investigations. The pathways involving the uptake, biosynthesis, excretion, and storage of cholesterol play a pivotal role in the initiation and progression of lung cancer. Despite a positive correlation between higher serum cholesterol levels and a lower incidence rate of lung cancer, the cholesterol levels within lung tumor cells have been found to be significantly elevated compared to adjacent normal cells. Strategies aimed at reducing cholesterol levels in tumor cells, such as inhibiting cholesterol uptake or biosynthesis and promoting excretion, have demonstrated the potential to decrease the incidence and progression of lung cancer. Additionally, the modulation of molecules along cholesterol metabolism pathways and metabolites can exert an influence on the growth and proliferation of lung cancer cells. In the systemic environment of lung cancer, cholesterol metabolism can also impact the functionality of immune cells in TME. While there is abundant evidence concerning the impact of cholesterol metabolism on lung cancer, details of the exact function of these effects and clinical applications are not fully demonstrated. Based on these findings, researchers are encouraged to further investigate more cholesterol metabolic targets to dysregulate the lipid balance in lung cancer cells, as well as the preclinical and clinical utility of these components in lung cancer. A growing body of evidence suggests that drugs targeting the cholesterol metabolism process have the capability to inhibit the progress of lung cancer. It is possible to develop more effective drugs based on cholesterol metabolism in tumor cells, and their clinical applications could be advantageous as lung cancer treatments. Moreover, as tumor cells constantly interact with TME, further investigations on cholesterol levels in TME, and targeted therapies, such as limiting cholesterol accumulation in TME, could be clinically beneficial.

Collectively, a deeper understanding of cholesterol metabolism and its effects on both tumor cells and immune cells within the TME could pave the way for innovative metabolic therapies to combat cancer.

## Data Availability

The datasets used and/or analyzed during the current study are available from the corresponding author upon reasonable request.
